# Isolation and genome annotation of Rowlf, a novel cluster EG bacteriophage with a purple acid phosphatase-like gene

**DOI:** 10.1128/mra.00314-25

**Published:** 2025-07-22

**Authors:** Emily Sullivan, Ninahazwe Mireill, Aisley B. Allen, Phoenix R. Larsen, Daniel Puentes Navarro, Madison P. Renn, Trenton W. Shappee, Madalyne M. Sisk, Alexander W. Thomas, Giselle Trejo, Lexy Bellew, Lee George, Sariah Hepworth, Savannah Webb, Sara Sadeghi, Joshua Burger, Eva Githuku, Jack F. Shurley, Anna S. Grinath, Michael A. Thomas

**Affiliations:** 1 Department of Biological Sciences, Idaho State University 6640, Pocatello, Idaho, USA; Queens College Department of Biology, Queens, New York, USA

**Keywords:** bacteriophage, *Microbacterium*, SEA-PHAGES, purple acid phosphatase

## Abstract

Isolated from grass thatch in Pocatello, Idaho, *Microbacterium foliorum* phage Rowlf has a siphovirus morphology and is presumed to be a lytic bacteriophage. Rowlf has a 62,044 bp genome with 104 putative genes, including a purple acid phosphatase-like gene found in leguminous plants and bacteria. Based on similarity to actinobacteriophages, Rowlf was assigned cluster EG.

## ANNOUNCEMENT

**Fig 1:**
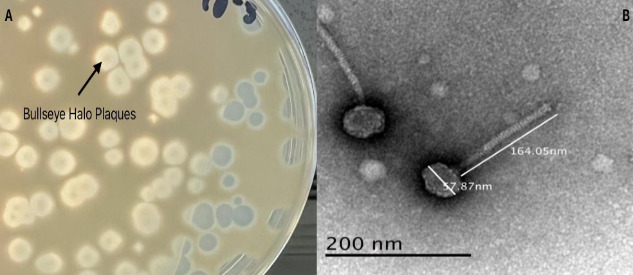
Plaque and particle morphology. (**A**) Rowlf forms plaques with a turbid center (see arrow) and halo. (**B**) Transmission electron micrograph reveals Rowlf to have a siphoviral morphology, with a long tail and icosahedral capsid. TEM measurements put the capsid diameter at 57–58 nm and tail length at 164–165 nm. Image produced by a Zeiss EM900 with an accelerating voltage of 80 kV and uranyl acetate negative staining; magnification was 140,000×; 30–40 virions were measured.

Bacteriophage isolation and characterization are important for advancing strategies to control bacterial growth, discovery of novel genes, such as biofilm depolymerase, and deploying phages as therapeutics (1–3). We describe the characteristics of a bacteriophage, Rowlf, isolated from thatch on Idaho State University’s campus in Pocatello, Idaho (42.867333°N, 112.429111°W). Utilizing established procedures (4–6), the sample was rinsed with 30 mL PYCa liquid media, centrifuged at 2,000×*g* for 10 min, and the supernatant filtered using a 0.22 µm filter. Filtrate was inoculated with *Microbacterium foliorum* NRRL B-24224, then incubated with shaking at 28°C for 48 h. An aliquot of the culture was then filtered and plated with PYCa top agar with *M. foliorum* and incubated at 28°C for 48 h. Plaques were purified with three additional rounds of plating and appeared medium in size with halo and bullseye (Fig. 1A). Uranyl acetate stain transmission electron microscopy revealed siphovirus morphology (Fig. 1B).

Rowlf DNA was extracted from high-titer (> 10^10^ pfu/mL) lysate using the Promega Wizard DNA cleanup kit and prepared for sequencing using a NEBNext Ultra II FS kit. DNA was sequenced using an Illumina MiSeq (v3 reagents) with a shotgun sequencing approach, generating 625,546 single-end 150-base reads with 1,512-fold coverage. Raw reads were assembled using Newbler v2.9 using default parameters. Consed v29 was used to check for completeness, accuracy, and determine phage termini, on default settings (7–9). The resulting genome was 62,044 bp with a direct terminal repeat of 199 bp and a GC content of 66.8%, similar to the host at 68.7% (10).

Rowlf’s genome was auto-annotated using DNAmaster v5.23.2 (https://phagesdb.org/DNAMaster/) embedded with Genemark v2.5 (11), Glimmer v3.02 (12), with subsequent manual review and confirmation. Phamerator v556 (13) was used to review synteny with other previously annotated genomes, and Starterator (http://phages.wustl.edu/starterator/) and Genemark v2.5p were used to review start site conservation and coding potential for each gene, respectively. HHpred v2.1 (14) searches of PDB, pfamA, and NCBI CD databases, and BLAST v2.15.0 (15) searches of PhagesDB (16) and NCBI non-redundant protein databases, were used to predict gene functions. The topmost GenBank blastn hit was phage Mashley (MN183284). There were no tRNAs detected by Aragorn v1.2.41 (17) and tRNA-SE v2.0 (18). Using default parameters for all software, 104 protein-coding genes were identified, and 40 were assigned putative functions. Rowlf was assigned to cluster EG based on similarity of gene content and order relative to other EG phages in the Actinobacteriophage database (19).

Consistent with other EG phages, a capsid maturation protease and minor capsid protein could be identified in Rowlf. One of Rowlf’s genes (PAP, gp26) shares structural similarity with purple acid phosphatase (PAP); using HHpred, the probability was >99% over >60% of the sequence relative to PAP of *Phaseolus vulgaris* (French bean; pdb1XZW). PAP genes are frequently observed in leguminous plants and occasionally in bacteria, where they appear to be used to prevent damage caused by oxygen reactivity (20). The function of a PAP-like gene in a phage remains unknown. Outside of Rowlf, only cluster EK phages are known to have PAPs.

## Data Availability

GeneBank accession and Sequence Read Archive (SRA) numbers are PP978829 and SRR27983390, respectively, at https://www.ncbi.nlm.nih.gov/.
